# Ten simple rules for initial data analysis

**DOI:** 10.1371/journal.pcbi.1009819

**Published:** 2022-02-24

**Authors:** Mark Baillie, Saskia le Cessie, Carsten Oliver Schmidt, Lara Lusa, Marianne Huebner, for the Topic Group “Initial Data Analysis” of the STRATOS Initiative

**Affiliations:** 1 Novartis, Basel, Switzerland; 2 Department of Clinical Epidemiology and Department of Biomedical Data Sciences, Leiden University Medical Center, Leiden, Netherlands; 3 Institute for Community Medicine, SHIP-KEF University Medicine of Greifswald, Greifswald, Germany; 4 Department of Mathematics, Faculty of Mathematics, Natural Sciences and Information Technology, University of Primorska, Koper, Slovenia; 5 Department of Statistics and Probability, Michigan State University, East Lansing, Michigan, United States of America

## Introduction

Data is the new oil [1], and analyzing data has been described as the number 1 profession of the 21st century [2]. But an appropriate analysis of data is also one of the most challenging tasks—a lot can go wrong at any step [3]. Researchers should always remember, but often forget, that data are numbers with context. When properties and context are not appropriately taken into account, data can speak through lies and riddles as preconditions for meaningful statistics are not met, often leading to harm [4–6].

Initial data analysis (IDA) provides a framework for researchers to work with data responsibly [4,7]. IDA has the following phases: (1) metadata setup; (2) data cleaning; (3) data screening; (4) initial data reporting; (5) refining and updating the research analysis plan; and (6) documenting and reporting IDA. These phases are core activities for all researchers who analyze data for primary or secondary use, e.g., data analysis of designed experiments, observational studies, patient registries, biobanks, or biomedical databases. Indeed, IDA can be described as the first data analysis step—the step to check if the observed data correspond to expectations about these data. Typically, researchers do not perform IDA in a systematic way, if at all, or mix IDA activities with subsequent data analysis tasks such as hypothesis generation or exploration, formal analysis, and interpretation of conclusions. As a consequence, researchers have many “degrees of freedom” [8], or may miss the “gorilla in the room” [9]. However, disciplined and systematic IDA practice can provide researchers with the necessary context about data properties and structures to avoid pitfalls [10].

IDA is often mistaken for exploratory data analysis (EDA) [11] as they share a large toolbox, including modern data visualization [12], but the aims are different. While EDA is a hypothesis-generating activity, IDA primarily ensures transparency and integrity of preconditions to conduct appropriate statistical analyses in a responsible manner to answer predefined research questions. For this purpose, IDA provides data ready for analysis including reliable information on the data properties. IDA could be likened to studying for an exam, reviewing materials, making sure the background is understood, and only then the “exam” is taken—the final analysis. The latter corresponds to executing the statistical analysis plan (SAP).

We have developed 10 rules to explain IDA and the benefits of adopting IDA in practice. The 10 rules are based on extensive experience with research projects, collaborations with domain experts, and discussions among an international group of applied statisticians. However, an understanding of IDA is important not only for statisticians but for all researchers who analyze data (e.g., epidemiologists, computational biologists, bioinformaticians, machine learning experts) or consume the outputs of data analysis (e.g., domain experts). These rules are applicable for small and large collaborative research projects, whether for primary data collection or repurposing an existing data set, and for data of all shapes and sizes including “big data.” A caveat for these 10 rules is that IDA is not an off-the-shelf cookbook. As with all good practice, IDA strategies require careful thinking and design based on the problem and context, not the preference of the analyst. Remember, “there are no routine statistical questions, only questionable statistical routines (David R. Cox)” (quoted in [10]).

### Rule 1: Develop an IDA plan that supports the research objective

During the course of a project researchers may perform additional ad hoc activities to help understand and interpret the findings of a key analysis. These activities are part of data cleaning or screening phases of IDA and may include assessing the distribution and scale of explanatory variables, checking for unusual or implausible observations, summarizing missing data patterns, or investigating any other data facets that could influence the formal analysis. The findings from these activities can then inform next steps such as model refinement or a change to the SAP. Decisions must be made to move the research forward. However, by not prospectively planning to assess data quality and context, the boundaries between answering the research question, model checking, data cleaning, or examining data properties can erode and turn into a cyclical investigation. This can place a huge, often hidden, cost on a project due to repeated analysis steps and, especially, a large communication overhead in the research team. Additionally, any data driven decision that deviates from the specified planned analysis, due to the back and forth between data checking and model development, risks the integrity and transparency of the project, especially if these activities are not clearly documented and reported [13].

Developing an IDA plan in conjunction with the study aims, design, and a (preplanned) SAP can manage this scope. Shortcomings in these first steps may result in forging ahead with inappropriate statistical methods or arriving at incorrect conclusions. The value of an effective IDA strategy for researchers lies in ensuring that data are of sufficient quality, that model assumptions made in the SAP are satisfied, or to support decisions for the statistical analyses (and are adequately documented). Asking questions at this initial stage of analysis can avoid headaches later when interpreting model results [14]. Planning for IDA can also ensure that activities related to data cleaning, checking, and examination are clearly accounted for, ensuring that enough time and resources are allocated, which leads to rule number 2.

### Rule 2: IDA takes time and resources

To ensure the integrity of a research project, IDA should be a key part of any research proposal. Do not underestimate the challenge of an effective IDA to conduct all necessary phases, but also do not underestimate the return on this investment. Researchers can expect to spend 50% to 80% of their time on the appropriate setup and understanding of data before the final analyses. These activities include setting up metadata, data cleaning, data screening, and documenting—in other words, the IDA phases. IDA requires a proper assignment of responsibilities. Factoring in time and resources into the research budget may seem like an additional upfront cost and burden, but not doing so can result in a more expensive cost later. A project that builds in sufficient time for planning, execution, and review of IDA can ensure that these tasks are performed systematically and by appropriate experts. IDA requires domain knowledge, especially researchers with an understanding of why and how the data was measured and collected, expertise in data management and stewardship, competencies in planning and implementing data analysis, and experience of scientific computing practices. The mixture of these competencies will be project specific, but also driven by the availability of resources. It can be a challenge to prospectively budget for IDA due to a lack of resources or organizational barriers. For example, some organizations may have a team of analysts who routinely perform IDA, while at other places, these are done by a single investigator who also handles the main statistical analyses [7].

### Rule 3: Make IDA reproducible

IDA is a crucial part of the research pipeline, and as such, it should be well documented to promote transparency, utility, and reproducibility. Therefore, keeping track of changes that you and your collaborators make to project data, programs (including analysis scripts, libraries, and packages), and documentation (including plans and reports) is a key IDA practice [15].

To make data reusable and data cleaning reproducible, the golden rule is to not change or overwrite the source data and organize the project so there is a clear distinction between source and derived data sets. All derivations from the source data, including the lineage of new variables and the rationale for the derivation, should be documented with a clear description of all the data cleaning rules. The rules should be implemented using computer code rather than manual changes to implement the rules. This is particularly important when multiple research team members have access to the data and can make modifications. Coding all the relevant rules can be challenging, but it will save a lot of time in the long term. Having well-documented metadata, making data cleaning code-based and repeatable, greatly enhances other people’s understanding of the data properties and data quality—including a future version of yourself who may need to revisit a project later. Take advantage of literate programming to reproducibly perform and document data screening. For example, document markup languages such as R markdown (https://rmarkdown.rstudio.com/) or Jupyter Notebook (https://jupyter.org/) serve the purpose, as they easily integrate narrative text and outputs from statistical analysis. A version control of the scripts [16] and automatization of steps will save a lot of time at second use, for example, when major revisions or additions are required. By ensuring the provenance and lineage of all analyses, any specific analyses can be traced back, rerun or revised and reproduced.

### Rule 4: Context matters: Know your data

IDA requires an understanding of what the numbers represent. This comprises the characteristics of the data, supplemented with background domain knowledge, how they were generated, and its intended use. Such knowledge is captured by the term metadata, or, in other words, data that describes other data. Metadata comprises, first, technical variable-related information such as labels, plausibility limits, codes for missing data, measurement units, or expectations about distributional properties and associations [17]. Such information is often available from data dictionaries. If not, it should be set up in a systematic way [18]. Second, it includes study and process level information related to the design characteristics of a data collection, recruitment of participants, and methods of performing measurements. Most of this information will commonly be available from the study protocol. If existing data are used, the source of the data needs to be clear. Third, metadata comprises knowledge about the intended analyses, which may be taken from an analysis plan. Such metadata will guide subsequent steps of the IDA process in a transparent manner. Set up all relevant metadata in an electronic form to facilitate its use during any statistical assessments [17].

Projects should include the setup, management, and publishing of metadata within a data management and stewardship plan [19]. These activities not only help researchers understand what the numbers represent but also ensure the reusability of data beyond the lifecycle of the project. By publishing metadata and IDA reports, researchers enable semantic interoperability—the exchange of data with unambiguous meaning and interpretation. These activities also encourage positive feedback loops such as future research building upon existing data resources.

### Rule 5: Avoid sneak peeks—IDA does not touch the research question

A good understanding of the research question, the intended analysis, and the data are all required to execute an analysis adequately and correctly interpret the findings. Matching the relevance of the data collection to the research question has been termed the “zeroth problem” [20]. IDA is a crucial step toward providing an analysis-ready dataset including reliable information about the data properties to answer the research question. However, a key IDA principle is not to touch the research question: Performing IDA to identify interesting patterns runs the risk of a data-driven selection of analyses and methods, chance observations, which might lead to incorrect or inflated claims. Choices may include unjustified removal of “undesired” observations or “optimizing” analysis strategies when performed unsystematically or separated from the scientific questions. It is important to avoid Hypothesizing After Results are Known, or HARKing, a term used for presenting results as having been hypothesized in advance, although they have not [21].

It may happen that IDA findings have consequences for the preplanned analyses. Parts of the original research plan may not be feasible due to data properties revealed by IDA, and IDA findings may require changes in the SAP. For example, skewed distributions may lead to applying a transformation, sparse multivariate distributions may identify the need to include or drop an interaction term, and certain observed missing data patterns may require more advanced methods to deal with missing data. All changes to the SAP should be well motivated, and well documented, which is more likely by separating IDA from the analyses performed to answer the research question.

### Rule 6: Visualize your data

The use of appropriate statistical graphics is a key IDA practice. Data visualization supports information seeking, pattern identification, and recognition during the phases of IDA. Visual displays ensure that relevant information (concepts, assumptions, patterns, trends, signals, and conclusions) is clearly presented and easy to interpret. This is especially useful when faced with high-dimensional data with many observations and measurement variables. Well-designed visual reports provide an “overview first, zoom and filter, then details-on-demand” [22], thereby supporting identification of emerging signals not in alignment with assumptions about the data and that could influence the interpretation of analysis results. It is important to be clear on the purpose and to be accurate with the implementation. Applying principles of effective visualization [23] is more than “plotting the data”; it can lead to a deeper understanding, while poor practice can lead to overlooking important context and to problems further in the research pipeline [3]. For example, visualizations designed for IDA help researchers identify important data properties that could be overlooked when using statistical graphics designed for later analysis phases [8]. A lot of ground has been covered on the topic of statistical graphics including flexible tools to support statistical graphics [12].

### Rule 7: Check for what is missing

Missing data are common in many research studies and are often not handled properly [24]. It can result in a reduction of the statistical power or can introduce important biases in the results. As a first step, investigate the frequency and proportion of missing values for each variable. Some observations may be missing by design; if available, the reasons for missingness coded in the metadata will shed light on this. As a second step, summarize how variables are missing simultaneously. Possible mechanisms for missingness can be investigated by comparing the subjects with missing and nonmissing values. A SAP should specify how missing data will be handled based on assumptions regarding the expected mechanism behind it (e.g., Rule 4, context matters). IDA can be used to reveal the potential impact of missingness and evaluate the appropriateness of the expected mechanism and the intended choices to handle missing data specified in the SAP. For example, if the proportion of missing values in an important explanatory variable is too large, or when individuals with missing values are considerably different than others, ignoring the missingness in subsequent statistical analysis is not appropriate. Guidance about handling missing data is available [24,25].

### Rule 8: Communicate the findings and consider the consequences

An IDA report should contain sufficient information that enables the research team to continue with the SAP in a responsible manner. It may include (1) a summary of the meta data, including the study design, the data sources, and the data content; (2) a study flow diagram illustrating sample size, inclusion and exclusion criteria, and subsequent selections being made to arrive at the sample size selected for the analysis; (3) a summary of the data cleaning process with an overview of data quality issues and an overview of rules used to identify and correct errors; (4) a description of the frequency and patterns of missingness; (5) an overview of univariate and multivariate distributions; (6) a summary of the findings that may influence the interpretation of the results; and (7) a summary of the findings, which may result in an update or refinement of the SAP.

During this process, domain expertise may be useful to understand deviations from expectations about the distributions of variables or what variables may be correlated. For example, the data screening could reveal an overrepresentation of older or higher educated people. In that case, there may be selection bias and domain expertise may be needed to select appropriate statistical countermeasures. Informed by the findings of IDA, researchers can prospectively plan appropriate sensitivity analyses to check the robustness of models or conclusions instead of relying on unexpected post hoc investigations [13].

### Rule 9: Report IDA findings in research papers

A clear and sufficient reporting of IDA findings in research papers is relevant for transparent and reproducible research. Yet, existing reporting guidelines [26] need to be augmented to accommodate findings specific to initial data analyses [13]. Importantly, IDA information that can influence the interpretation of the results, or IDA findings that have led to adjustments in the analysis plan, should always be mentioned in a comprehensive and systematic way. For example, methods or decision rules for data preprocessing and possible changes in the analysis plan should be described in the methods section of a paper. A flow chart of the study inclusion and a table describing important variables with numbers of missing values should be placed in the results section, while IDA findings, which influence the interpretation of results, could be summarized in the discussion part or limitations.

### Rule 10: Be proactive and rigorous

It is much better to avoid data issues right at the beginning of a data collection than to search for statistical workarounds thereafter [14]. Keeping this in mind, researchers should closely cooperate on the integrity of a study during its design and conduct stage. This encompasses an appropriate, structured setup of metadata before the start of the data collection to identify design flaws. Appropriate metadata setup is a precondition for efficient monitoring processes. Conducting selected IDA steps as part of a data monitoring at regular intervals during study conduct will facilitate the early detection and mitigation of data quality issues.

## Conclusions

IDA is a common task for all quantitative research. To ensure the integrity of a research project, IDA should be a key part of any research proposal. Do not underestimate the challenge of an effective IDA to conduct all necessary phases, but also do not underestimate the return on this investment. It is a crucial step and aims for transparency and integrity by providing you with an analysis-ready data set and reliable information about its properties that enables you to perform the statistical analyses in a responsible manner and interpret the obtained results. Developing an a priori IDA plan allows you to perform IDA in an efficient and fast manner including the necessary elements to communicate the findings to the research team and in a publication, as well as avoiding “pitfalls.” Following these rules can also help future researchers reliably reuse your data and research outputs, by making the often hidden decisions of data analysis more transparent, by helping to separate the phases of IDA from the final data analysis, and by publishing all relevant research materials including metadata, code, and IDA reports.
